# Alert on a Novel South American Bat Coronavirus with a Furin-Cleavage Site: A One Health Early Warning

**DOI:** 10.1590/0037-8682-0583-2025

**Published:** 2026-06-15

**Authors:** Lucas Alves da Mota Santana, Lara Góis Floresta, Êmilly Victória Maciel Alves, Breno Ferreira Barbosa, Graziane Ribeiro Couto, Marcos Antônio Lima dos Santos, Pedro Henrique Macedo Moura, Deise Maria Rego Rodrigues Silva, Marina dos Santos Barreto, Carlos Eduardo Palanch Repeke, Regiane Cristina do Amaral, Virgínia Kelma dos Santos Silva, Cleverson Luciano Trento, Wilton Mitsunari Takeshita, Dalmo Correia, Rajiv Gandhi Gopalsamy, Lysandro Pinto Borges

**Affiliations:** 1 Universidade Federal de Sergipe, Departamento de Odontologia, Aracaju, SE, Brasil.; 2 Universidade Tiradentes, Departamento de Odontologia, Aracaju, SE, Brasil.; 3 Universidade de São Paulo, Faculdade de Odontologia, Departamento de Estomatologia, São Paulo, SP, Brasil.; 4 Universidade Federal de Sergipe, Departamento de Farmácia, São Cristóvão, SE, Brasil.; 5 Universidade Federal de Sergipe, Departamento de Odontologia, Lagarto, SE, Brasil.; 6 Universidade Estadual Paulista Júlio de Mesquita Filho, Faculdade de Odontologia, Departamento de Diagnóstico e Cirurgia, Araçatuba, SP, Brasil.; 7 Universidade Federal de Sergipe, Departamento de Medicina, Aracaju, SE, Brasil.; 8 Universidade Tiradentes, Departamento de Medicina, Aracaju, SE, Brasil.; 9 Rajagiri College of Social Sciences, Division of Phytochemistry and Drug Design, Department of Biosciences, Kochi, Kerala, India.

Dear Editor:

The recent discovery of a bat-related zoonotic (BRZ) batCoV beta coronavirus in the Neotropical bat *Pteronotus parnellii* has renewed concerns about coronavirus evolution and zoonotic risk. The COVID-19 crisis demonstrated how coronaviruses, especially those of bat origin, can cross species barriers and generate sustained global public health consequences^1^. Coronaviruses are positive-sense, single-stranded RNA viruses capable of causing a wide spectrum of disease, ranging from mild respiratory infections to severe and fatal pneumonia across multiple host species^2^.

An interesting finding is the presence of an active furin cleavage site (FCS) at the S1/S2 junction of the spike (S) protein of BRZ batCoV, which has been shown to increase viral infectivity and cell entry^3^. The key viral entry determinant of the spike (S) protein consists of two functional homotrimeric subunits, S1 and S2. The S1 subunit hosts the receptor-binding domain responsible for viral attachment to host cells, while the S2 subunit hosts the fusion peptide that triggers fusion of the virus into the host cell following interaction with the ACE2 receptor^4^.

According to Shanmugaraj et al. (2026)^3^, the discovery of an FCS-containing coronavirus with a polybasic furin cleavage site (RRAR) challenges the previous assumption that these were restricted to Old World lineages. Moreover, RRAR in SARS- CoV-2 enables spike activation via host proteases, which enhances fusogenicity, viral entry, and syncytium formation^5^. Proteolytic cleavages at the S1 and S2 sites by host proteases, such as TMPRSS2, cathepsin L, trypsin, and furin, have a strong effect on viral pathogenicity and tropism^6^. The biological significance of FCS deletion is further supported by loss-of-function experiments, which show a significant decrease in viral growth and virulence in vivo^7^.

 Hernandez-Aguillar et al. (2021)^8^ identified a significant knowledge gap in regional surveillance by conducting widespread monitoring throughout the Americas; more than 180 bat coronaviruses across 43 species in the Americas were already known but none of them had FCS. Further research by Ruiz-Aravena et al. (2022)^9^ showed that mining, deforestation, and habitat degradation intensify the level of stress amongst bats, increasing viral shedding and opportunities for human exposure. In this context, finding BRZ batCoV in an ecologically degraded yet biodiverse region supports the need for a coherent One Health approach that integrates human seroepidemiology, genomic surveillance, and animal ecology into a single system. 

Assessment of the zoonotic potential of BRZ batCoV requires evaluation of both translational and biosafety aspects. Functional characterization should include analysis of ACE2 binding affinity, replication efficacy in airway organoids and susceptibility to neutralization, all conducted under BSL-3 conditions and within ethically sound wildlife research frameworks^10^. Importantly, furin cleavage sites have a natural evolutionary origin at the S1/S2 junction with the presence of amino-acid insertions observed in bat coronaviruses such as RmYN02 and Bat CoV CD35^11^. Phylogenetic and genomic evidence indicates that Bat CoV CD35 is a basal SARS-associated coronavirus belonging to the subgenus Hibecovirus and represents an immediate ancestor of Bat Hp-2CoV. Unlike other bat SARS-related viruses, CD35 has a polybasic, furin-like S1/S2 cleavage site with a high cleavage score, comparable to those observed in SARS -CoV-2 and MERS-CoV^5^. 

These studies underline the need for continued coronavirus surveillance in wildlife and the importance of maintaining strict biosafety (including BSL-3 containment), as well as adherence to the ethical standards governing animal research. A coordinated strategy involving academic researchers, veterinarians, and public health authorities, alongside transparency and the sharing of data, is crucial to reduce speculation and fake news about the origin of viruses^12^.

The absence of a polybasic FCS at S1/S2 junction of the S-protein in existing coronaviruses has been associated with lower infectivity and milder symptoms. In contrast, the presence of BRZ batCoV, which has a functional furin cleavage site, can facilitate spike activation, increase viral entry, and broaden host and tissue tropism. While this characteristic is linked to higher pathogenicity, no evidence of human infection or transmission of BRZ batCoV has been recorded to date. Thus, the threat posed by BRZ batCoV to human health remains speculative, further emphasizing the importance of monitoring this virus and engaging in functional research, rather than focusing on current human impact.

In conclusion, the identification of a bat coronavirus containing an FCS in Brazil broadens the range of potentially zoonotic beta coronaviruses and reinforces South America's importance in terms of future pandemic preparedness research. Although human infection has not yet been demonstrated, strict genetic and ecological monitoring remains warranted. Therefore, this discovery is a timely reminder of the need to invest in regional virome surveillance and to strengthen cross-disciplinary One Health capacity as a long-term global health priority.
